# Digital Mental Health Interventions for Depression, Anxiety, and Enhancement of Psychological Well-Being Among College Students: Systematic Review

**DOI:** 10.2196/12869

**Published:** 2019-07-22

**Authors:** Emily G Lattie, Elizabeth C Adkins, Nathan Winquist, Colleen Stiles-Shields, Q Eileen Wafford, Andrea K Graham

**Affiliations:** 1 Center for Behavioral Intervention Technologies Northwestern University Chicago, IL United States; 2 Department of Psychiatry and Behavioral Sciences Rush University Medical Center Chicago, IL United States; 3 Galter Health Sciences Library and Learning Center Northwestern University Chicago, IL United States

**Keywords:** eHealth, mHealth, mental health, students, universities

## Abstract

**Background:**

College students are increasingly reporting common mental health problems, such as depression and anxiety, and they frequently encounter barriers to seeking traditional mental health treatments. Digital mental health interventions, such as those delivered via the Web and apps, offer the potential to improve access to mental health treatment.

**Objective:**

This study aimed to review the literature on digital mental health interventions focused on depression, anxiety, and enhancement of psychological well-being among samples of college students to identify the effectiveness, usability, acceptability, uptake, and adoption of such programs.

**Methods:**

We conducted a systematic review using the Preferred Reporting Items for Systematic Reviews and Meta-Analyses guidelines (registration number CRD42018092800), and the search strategy was conducted by a medical research librarian in the following databases: MEDLINE (Ovid), EMBASE (Elsevier), PsycINFO (EbscoHost), the Cochrane Library (Wiley), and Web of Science (Thomson Reuters) from the date of inception to April 2019. Data were synthesized using a systematic narrative synthesis framework, and formal quality assessments were conducted to address the risk of bias.

**Results:**

A total of 89 studies met the inclusion criteria. The majority of interventions (71/89, 80%) were delivered via a website, and the most common intervention was internet-based cognitive behavioral therapy (28, 31%). Many programs (33, 37%) featured human support in the form of coaching. The majority of programs were either effective (42, 47%) or partially effective (30, 34%) in producing beneficial changes in the main psychological outcome variables. Approximately half of the studies (45, 51%) did not present any usability or acceptability outcomes, and few studies (4, 4%) examined a broad implementation of digital mental health interventions on college campuses. Quality assessments revealed a moderate-to-severe risk of bias in many of the studies.

**Conclusions:**

Results suggest that digital mental health interventions can be effective for improving depression, anxiety, and psychological well-being among college students, but more rigorous studies are needed to ascertain the effective elements of these interventions. Continued research on improving the user experience of, and thus user engagement with, these programs appears vital for the sustainable implementation of digital mental health interventions on college campuses.

## Introduction

In the last decade, rising rates of college students experiencing symptoms of depression and anxiety have been observed [1-3]. Globally, approximately 31% of college students screened positive for a mental health disorder over the course of the last year [4]. It has also been increasingly recognized that accessing treatment for these common mental health problems is difficult. Many students have low mental health literacy and do not recognize a need for treatment but rather believe that these depression and anxiety symptoms are typical college stress and, therefore, do not warrant treatment [5]. Students who do recognize a need for mental health services often face multiple barriers to accessing care, perceive the care available to them as inconvenient, and are skeptical about the efficacy of care [6,7].

Campus counseling centers are well positioned to provide mental health care. However, many counseling centers across the country are underresourced, have difficulty reaching students in need, and operate at full capacity during much of the year [8].

Digital mental health interventions, such as those delivered via mobile- and Web-based platforms, offer the possibility of treatment to college students with common mental health problems while circumventing many existing barriers to receiving traditional mental health services, including stigma and time [9-11].

The evidence base for digital mental health interventions for general adult populations is vast [12-15], and the evidence base for college and university student populations is rapidly accumulating. In 2013, a systematic review of technology-based interventions for mental health in tertiary students found that these types of interventions offer promise for improving symptoms of certain mental health problems, but it concluded that more research was needed [16]. A 2014 systematic review and meta-analysis of computer-delivered and Web-based interventions for university students found that these types of interventions can be effective in improving depression, anxiety, and stress among students [17]. More recently, a 2018 systematic review and meta-analysis found that internet interventions can have small-to-moderate effects on a range of mental health conditions [18].

However, there have been limitations of these past reviews, as they have focused exclusively on studies that were randomized controlled trials (RCTs). Although a focus on studies with RCT designs allows researchers to evaluate the efficacy and effectiveness of digital mental health interventions, the exclusion of papers reporting on other study designs presents a significant gap in our ability to assess the uptake and adoption of digital mental health interventions for university students (which could be assessed in nonrandomized designs, including single-arm trials in which an intervention is made available to all students on campus). This is particularly important as the full public health potential of these types of interventions is tied not only to clinical efficacy but also to the successful implementation of these programs in real-world settings. Across the board, the implementation and integration of digital health tools into routine care settings has been a challenge. Many have called for testing digital health tools under more pragmatic conditions to maximize the transfer of knowledge from research trials to real-world implementation [19-21], and studies examining the real-world uptake and engagement with digital mental health tools have generally found low engagement and completion rates [22]. Furthermore, in recent years, increased focus has been on assessing the user experience (including the usability and acceptability) of such interventions to identify and rectify user experience failings that could limit one’s ability and desire to continue to use a program [23-25]. The aim of this systematic review was to evaluate the effectiveness, usability, acceptability, uptake, and adoption of digital mental health interventions for treating depression and anxiety and for enhancing psychological well-being among college students. Characteristics of the student digital mental health interventions have been described here.

## Methods

### Eligibility Criteria

To be included in this review, studies had to (1) examine an intervention that aimed to improve psychological well-being, psychological distress, stress, depressive, and/or anxious symptoms; (2) deliver the intervention via a digital platform (including mobile phone, website, virtual reality systems, and offline computer programs; they could be delivered as an adjunct to face-to-face interventions); (3) include students enrolled in higher education institutions, such as 2-year community colleges, professional schools (eg, medical school and nursing school), 4-year colleges (ie, bachelor’s degree–granting institutions that do not offer graduate degrees), and universities; (4) report outcomes related to psychological well-being, psychological distress, stress, depressive and anxious symptoms, and/or the use and reach of an intervention; and (5) be written in English. In this paper, we use the term *college students* to refer to all students in postsecondary education, including medical students. All study designs were included, with the exception of technical validation papers reporting exclusively on the development of digital mental health interventions. Conference abstracts were also excluded.

### Search Strategy

A comprehensive search strategy was developed using keywords and controlled vocabulary to describe university students, depression and anxiety, and digital mental health interventions. The search strategy was adapted to the electronic databases MEDLINE (Ovid), EMBASE (Elsevier), PsycINFO (EBSCOhost), Web of Science (Thomson Reuters), and the Cochrane Library (Wiley). Each database was searched from the date of inception to April 18, 2019. As some relevant journals (ie, *JMIR Mental Health* and *Digital Health*) are not indexed in the searched sources, an additional handsearch was conducted through these publications and through the reference lists of related systematic reviews. The searches were not limited based on publication date, language, document type, or study design. Throughout the study selection, the reference lists of included studies were further reviewed to identify relevant citations. The search strategy terms are presented in Multimedia Appendix 1. The review adhered to the Preferred Reporting Items for Systematic Reviews and Meta-Analyses (PRISMA) guidelines [26] and was registered before data extraction on the international prospective register of systematic reviews PROSPERO website (registration number CRD42018092800).

### Study Selection

Search results were uploaded into Rayyan, a Web-based software program that allows for reviewers to collaborate during the study selection process [27]. Two reviewers independently screened each of the titles and abstracts from the initial literature search against the inclusion criteria. Authors EGL, ECA, NW, CSS, and AKG served as reviewers. Full-text articles for the approved articles were then screened independently by 2 reviewers. Discrepancies about inclusion were resolved by discussion, and a third reviewer was brought into the discussion if necessary.

### Data Extraction

Two reviewers extracted the data independently from each eligible study using a Web-based extraction form that was piloted and calibrated with all reviewers before formal data extraction. Discrepancies about data extraction were resolved by discussion, and a third reviewer was brought into the discussion if necessary. The data extracted included the study location, study design, type of comparator, type of prevention/treatment, type of technology, name of technology/program, type of program, primary intervention target(s), presence of support, student population, setting, sample size, length of intervention, usability and acceptability outcomes, uptake and adoption outcomes, psychological outcomes, and type of analyses performed (ie, completer or intent to treat).

### Outcomes

This review examined the effectiveness, usability, acceptability, uptake, and adoption of digital mental health interventions for treating depression and anxiety and for enhancing psychological well-being among college students. The effectiveness outcomes included measures of depressive symptomatology (eg, Beck Depression Inventory-II [28] and Patient Health Questionnaire [29]), measures of anxious symptomatology (eg, Beck Anxiety Inventory [30] and Anxiety Sensitivity Inventory [31]), and measures of psychological distress and well-being (eg, Perceived Stress Scale [32] and Scales of Psychological Well-being [33]).

For the purpose of this review, usability was defined as the quality of a user’s experience when interacting with a program. Usability is an umbrella term that includes the ease of learning a program, the efficiency of use, the memorability of it, and the subjective satisfaction with a program. The usability outcomes include standard usability measures (eg, the System Usability Scale [34]) and qualitative usability reports. Acceptability is specifically about satisfaction with different aspects of the program and was primarily measured through qualitative self-reporting.

For the purpose of this review, the terms *uptake* and *adoption* were used in conjunction with one another [35] and were together defined as the action of trying an innovation. Thus, uptake and adoption outcomes were primarily metrics on the number of downloads and uses and were intended to be described alongside the number of users relative to the population of potential users when available (to determine the rates of service penetration [35]). However, few studies provided these details. Metrics on the completion of follow-up assessments and study attrition (or fidelity to the intervention) were examined in studies that did not provide detailed program usage metrics to allow for further implementation-related insights to be gathered.

### Quality Assessment

As this review included both randomized trials and nonrandomized trials, the risk of bias was assessed using 2 separate tools: the Cochrane Collaboration’s tool for assessing risk of bias in randomized trials [36] and the Cochrane Collaboration’s tool for assessing risk in nonrandomized studies of interventions [37]. For randomized trials, risk of bias was evaluated for selection bias, performance bias, detection bias, attrition bias, and reporting bias using the anchors of a low, high, or unclear risk of bias. For nonrandomized trials, risk of bias was evaluated for bias because of confounding, bias in selection of participants into the study, bias in classification of interventions, bias because of deviations from intended interventions, bias because of missing data, bias in measurement of outcomes, and bias in selection of the reported result.

### Data Synthesis

A systematic narrative framework was used to synthesize the data [38-40]. Owing to the high degree of heterogeneity in outcomes and measurement included in this study, a meta-analytic approach was not appropriate. Following the systematic narrative framework for literature reviews [39], the results of included studies were synthesized and presented without reference to the statistical significance of the findings.

## Results

### Included Studies

**Figure 1:**
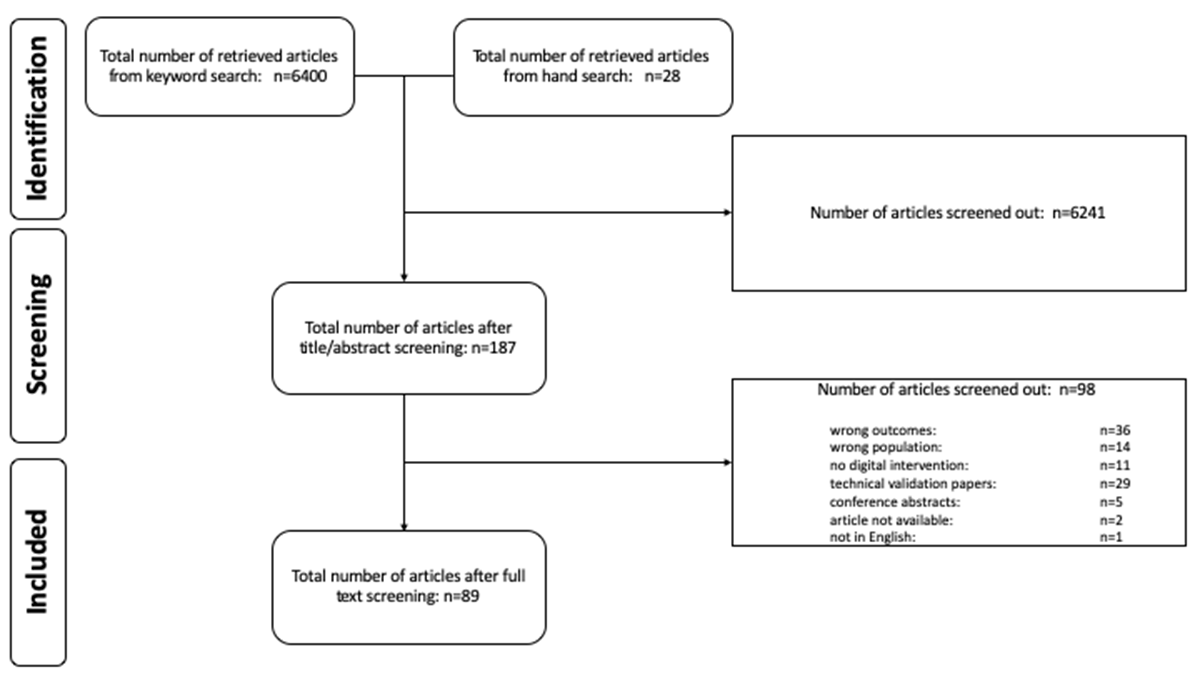
Preferred Reporting Items for Systematic Reviews and Meta-Analyses flow diagram.

A total of 6428 article titles and abstracts were reviewed. Then, 187 full-text articles were reviewed for inclusion, with 89 studies included in the review for data extraction. See Figure 1 for the PRISMA flow diagram.

### Study Characteristics

Of the 89 studies included in this review, 72 employed randomized study designs [41-112], whereas the remaining 17 were nonrandomized [113-129]. One of the included studies [83] was a secondary analysis of another included study [84]. Another one of the included studies [129] was a qualitative study of the usability and acceptability of another included study [101]. The vast majority of the included studies (n=81 [41-44,47-52,54-56,58-111,113,114,116-120,122,123,125-129]) took place at universities, with far fewer taking place at 4-year colleges (n=2 [45,46]), at health professional schools (eg, medical school and nursing school; n=5 [57,115,121,124,130]), and at community colleges (n=1 [53]). Approximately half of the studies (n=44 [42-48,50,53,55,56,58-61,63,65,66,72,73, 76-78,81,87-90,93,95,98,99,101-104,106,108,110,116-118, 122,125]) targeted undergraduate students exclusively. Most studies included were focused on either universal prevention programs (n=36 [43,48,53,54,63-67,70-73,75-78,90,91, 94,95,97,99,100,102,106-108,110-112,114,118,119,125,126]), or on treatment intervention programs (n=22 [41,49,50,52, 58,69,74,79,80,83-86,88,89,92,98,113,117,120,122,127]).

We examined if each study appeared to be designed specifically for students (eg, the purpose of the study was focused on college student mental health) or if college students appeared to be used as a convenience sample. As seen in Multimedia Appendix 2, a minority of the studies (n=12 [44,71,89,90,93,95,104, 110,111,114,118,123]) appeared to use college students as a convenience sample, and for an additional 9 studies, it was unclear if the program was being tested specifically for college student mental health or if college students were a convenience sample [47,50,51,61,64,68,84,105,122].

The majority of these studies (n=46 [43-48,51-55,57-59, 62,65-67,69,72,76-78,86,87,113,116,119,120,122,124-127,131]) took place in the United States. As seen in Multimedia Appendix 2, several studies also took place in the United Kingdom (n=6 [60,73,75,80,96,114]), Ireland (n=5 [83-85,117,123]), Australia (n=5 [50,61,74,109,115]), Canada (n=5 [42,49,81,102,103]), and China (n=5 [70,71,89,91,100]).

A total of 71 studies utilized a Web-based technology ([42-44, 46,49,50,53-55,57,59-88,90,91,94,96-101,103-110,112,113,115-121, 123-126,128]), and 8 studies utilized interventions delivered via mobile phone (app-based programs and short message service–based programs) [52,56,59,95,101,102,114,129]. Furthermore, 11 studies utilized offline computer-based programs [41,45,47,51,52,89,92,93,105,111,127], and 3 studies focused on virtual reality programs [58,111,122]. As seen in Multimedia Appendix 2, some studies included more than one type of technology, and thus, the numbers reported in the previous sentence add up to more than the 89 studies included in this review. Although the interventions examined were variable in content and in length, the most common type of intervention examined was internet-based cognitive behavioral therapy (n=28 [42,49,50,57,61,68,72-75,79-81,83-86,88,98,101, 103,107,109,112,113,123,128,129]). The modal length of intervention was 8 weeks. Most studies focused on digital mental health programs developed for the specific study, whereas a minority of studies (n=15) focused on publicly available interventions (ie, Beating the Blues, n=3 [83,84,86]; MoodGYM, n=5 ([50,57,68,98,112]); Therapist-Assisted Online, n=1 [113]; Headspace and Smiling Mind, n=1 [95]; Family eJournal, n=1 [90]; Tess chatbot, n=1 [97]; DeStressify, n=1 [102]; Overcome Social Anxiety, n=1 [103]); SilverCloud Health’s Space from Depression, Space from Anxiety, and Space from Stress, n=1 [128]; and CareCollaborateConnect: Student Success and thedesk, n=1 [109]).

The majority of interventions studied offered some level of support or guidance to users—many of these interventions featured coaching from a human (n=33 [41,49-51,58 ,62-66,69,71,73,74,76,79,82-88,94,101,104,105,107,116,122,123,128,129]), whereas others featured automated support (n=18 [44,48, 53,57,59,67,70,77,91,98-101,106,108,112,113,115]), such as prescripted emails. Several studies examined self-guided interventions (n=33 [42,43,45,46,52,54-56,60,61,68,72,75,78, 80,81,89,90,92,93,95,97,102,103,109-111,114,118,119,121,124,125]) in which the participants only had contact with the study staff for research assessments; few studies focused on technology as an adjunct to therapy (n=5 [47,86,100,120,126]), 2 studies provided peer support [96,117], and 1 study had an unclear presence of support (n=1 [127]).

### Effectiveness of Interventions Studied on Psychological Outcomes

As seen in Multimedia Appendix 3, the majority of studies reported that the digital mental health intervention(s) of interest were either effective (eg, improvements were observed in all main outcomes; n=42 [41,42,45,47-51,54,56,57,59,60,62,63, 68,71,78,79,84-89,92,93,100,101,103,112,113,115,120,122,123,125, 127,128,131]) or partially effective (eg, improvements were observed in some main outcomes; n=30 [43,44,52,53,58,61, 65,67,69,70,72-77,80-82,90,91,94-97,99,102,108, 111,124]) in producing beneficial changes in the main psychological outcome variables. A minority of studies reported on interventions that were not effective (n=10 [46,55,64,66,98,114,117]) or did not report on psychological outcomes and focused on program usage or usability (n=6 [116,118,119,121,126,129]). An examination of intervention effectiveness by type of technology used follows. Of the 71 studies that included a Web-based intervention, 30 were effective, 25 were partially effective, 8 were not effective at producing beneficial changes in the main psychological outcome variables, and for 8 studies, effectiveness was not applicable. Of the 8 studies that included interventions delivered via mobile phone, 3 were effective, 3 were partially effective, 1 was not effective, and for 1 study, effectiveness was not applicable. Of the 11 studies that included offline computer-based programs, 10 were effective and 1 was partially effective. Of the 3 studies that included a virtual reality–based intervention, 1 was effective and 2 were not effective.

Of the 42 studies deemed effective in producing beneficial changes in the main psychological outcome variables, 36 of those studies utilized a control condition; although the quality of control conditions varied broadly and ranged from treatment as usual or waitlists to other digital programs or face-to-face treatment [41,42,45,47,48,50,51,54,56,57,59,60,71,78,79,84, 86,88,89,92,93,100,101,103,105,107,109,112,113,125]. See Multimedia Appendix 2 for more information. Some studies targeted general mental health and well-being and listed several primary outcome variables; thus, it was difficult to ascertain whether the intervention was *fully* effective. To provide a conservative estimate, these studies were counted as partially effective.

### Usability and Acceptability of Interventions Studied

Approximately half of the studies included in this review (n=45 [41-44,48,54-57,59,60,62,63,70,73,76-78,81,84,85,87-93,96, 100,102,104,106,110,111-113,116,118,121-125,127]) did not present any usability or acceptability outcomes. In those studies that presented usability and acceptability outcomes, the results were generally favorable. However, response rates were often low (which was specifically noted in the studies by Lintvedt et al [68] and Mailey et al [69]), so it is difficult to ascertain the true acceptability of these interventions as those who continued to engage with the study procedures may have found the interventions more useful and usable than those who did not.

As seen in Multimedia Appendix 3, studies that presented usability and/or acceptability outcomes typically relied on single-item Likert scales, questionnaires, or user feedback interviews rather than validated measures. A minority of studies used validated measures such as the System Usability Scale [65-67,120,132] to assess usability and the Client Satisfaction Questionnaire [61,71,86,133] to assess acceptability. The results of formal usability testing were not presented in any of the studies.

### Uptake and Adoption of Interventions Studied

The vast majority of studies did not specify the size of the pool of potential participants from which the study participants were drawn (n=81 [41-54,56-61,63-71,73-91,93-101,103-105, 107-111,113-115,117-127,129]) and recruited from seemingly large pools of students. Furthermore, many studies did not present the metrics on the usage of the digital mental health interventions (n=29 [44,45,48,50,53,56,69,70,73,74,76-78,81, 89,90,93,98,99,103-105,107,109-111,113,114,124]. As detailed program usage metrics were not addressed by the authors of several included studies, the *Uptake and Adoption* column in Multimedia Appendix 3 includes additional data on completion of follow-up assessments and study attrition as a proxy for intervention uptake and adoption. For studies that did not provide clear metrics on the completion of follow-up assessments and/or study attrition, we have listed *not reported* in this column. For studies that examined digital mental health interventions in standardized laboratory settings, we have listed *N/A due to standardized within-lab use*.

Relatively few studies examined the implementation of a digital mental health intervention on a college campus and reported on the implementation outcomes [35,93,98]. Although a small handful of studies reported on the broad uptake and adoption of programs that were implemented on college campuses [86,116,119,128], the Beating the Blues implementation by Santucci et al [86] and the SilverCloud implementation by Palacios et al [128] were the only studies in which the feasibility of implementing a digital mental health program was explicitly discussed. Santucci et al set out to assess the feasibility, acceptability, and effectiveness of Beating the Blues for university students (as benchmarked to published trials) and outlined their process of conducting a needs assessment and engaging stakeholders before commencing the trial [86]. They found preliminary support for the feasibility of disseminating and implementing Beating the Blues in a university health center and effectiveness similar to what had been documented in a previous RCT. Palacios et al [128] conducted an open trial in which 3 SilverCloud programs—Space from Depression, Space from Anxiety, and Space from Stress—were made available to the students and were advertised through on-campus counseling centers. The majority of the participants found the programs helpful and found benefit in having a supported Web-based intervention available on campus, and the programs demonstrated feasibility, acceptability, and effectiveness.

As seen in Multimedia Appendix 3, many studies had high rates of attrition and low rates of sustained program use. Although usage was variable and cannot be directly compared across studies, a pattern emerged such that for module-based interventions, usage dropped over an individual’s time spent in the study. For example, module 1 program completion rates were generally high, and in many studies examined, a minority of participants completed all available modules.

### Risk of Bias

As seen in Table 1, of the 72 randomized studies, 28 studies were judged as having a low risk of bias ([41,44,47,54,57, 60,64,66,75,78,84,86,88,91,92,94,95,97,99,100,101,102-104, 109,111,112]) and only 9 studies were judged as having a high risk of bias [45,49,58,63,65,72,83,96,110]. The remaining 35 studies were judged as having some concerns regarding bias [42,43,46,48,50-53,55,56,59,61,62,67-71,73,74,76,77,79-82,85,87,89,90, 93,98,105-108,131]. Risk of bias most frequently emerged because of a potential bias in measurement of the outcome. Some concerns were noted in this domain for roughly one-third of the studies (n=27 [42,46,49,50,52,53,55,56,58,59,61-63, 65,67-70,72-74,76,81,82,85,87,89]) because outcomes were self-reported in nature and the participants were aware of the intervention they received. Concerns were often noted regarding potential bias arising from the randomization process, and potential bias because of missing outcome data that were not analyzed in a manner to minimize risk [80-82]. Potential bias arising from the randomization process was frequently noted because of baseline imbalances that suggested problems with randomization. Concerns surrounding risk of bias because of missing outcome data were frequently related to high levels of attrition, which is a particularly common problem in studies of digital mental health interventions.

Of the 16 nonrandomized studies screened for risk of bias, the majority of studies (n=10 [113-115,117,120,122-125,128]) demonstrated a serious risk of bias using the Risk of Bias in Non-Randomized Studies of Interventions (ROBINS-I) rating scale, and 1 study demonstrated a critical risk of bias [127]. One qualitative study [129] from which we extracted usability and acceptability data was not included in the risk-of-bias assessments. This study collected data from participants of an already analyzed RCT [101]. The details are provided in Table 2. Risk of bias is more common in nonrandomized studies, and the 3 studies that were determined to have a low risk of bias [116,118,119] all reported on the uptake and adoption of digital mental health programs as their main outcomes. As these metrics were objective, minimal risk of bias was identified. For the majority of studies, a potential for bias was identified in the measurement of outcomes, as outcomes were self-reported in nature and the participants were aware of the intervention they received.

**Table 1:** Risk of bias for randomized studies.

Authors and year of publication	Dmn^a^ #1^b^	Dmn #2^c^	Dmn #3^d^	Dmn #4^e^	Dmn #5^f^	Overall risk
Alvarez et al, 2008 [41]	LR^g^	LR	LR	LR	LR	LR
Arpin-Cribbie et al, 2012 [42]	LR	LR	LR	SC^h^	LR	SC
Asbury et al, 2018 [90]	SC	SC	LR	LR	LR	SC
Auyeung & Mo, 2018 [91]	LR	LR	LR	LR	LR	LR
Bedford et al, 2018 [92]	LR	LR	LR	LR	LR	LR
Booker & Dunsmore, 2017 [43]	LR	SC	SC	LR	LR	SC
Braithwaite & Fincham, 2009 [44]	LR	LR	LR	LR	LR	LR
Braithwaite & Fincham, 2007 [93]	SC	LR	SC	LR	LR	SC
Buglione et al, 1990 [45]	SC	SC	SC	LR	LR	HR^i^
Chiauzzi et al, 2008 [46]	LR	LR	LR	SC	LR	SC
Cohen et al, 1999 [47]	LR	LR	LR	LR	LR	LR
Cukrowicz & Joiner, 2007 [48]	SC	LR	LR	LR	LR	SC
Day et al, 2013 [49]	LR	LR	HR	SC	LR	HR
Ellis et al, 2011 [50]	LR	LR	LR	SC	LR	LR
Eustis et al, 2018 [94]	LR	LR	LR	LR	LR	LR
Fernandez et al, 1986 [51]	LR	LR	LR	LR	SC	SC
Fitzpatrick et al, 2017 [52]	LR	LR	LR	SC	LR	SC
Flett et al, 2019 [95]	LR	LR	LR	LR	LR	LR
Frazier et al, 2015 [53]	LR	LR	LR	SC	LR	SC
Freeman et al, 2008 [96]	SC	LR	HR	HR	LR	HR
Frith & Loprinzi, 2017 [54]	LR	LR	LR	LR	LR	LR
Fulmer et al, 2018 [97]	LR	LR	LR	LR	LR	LR
Geisner et al, 2015 [55]	LR	LR	LR	SC	LR	LR
Gibbel, 2010 [98]	LR	SC	LR	LR	LR	SC
Grassi et al, 2011 [56]	LR	LR	SC	SC	LR	SC
Greer, 2015 [99]	LR	LR	LR	LR	LR	LR
Guille et al, 2015 [57]	LR	LR	LR	LR	LR	LR
Hall et al, 2018 [100]	LR	LR	LR	LR	LR	LR
Harrer et al, 2018 [101]	LR	LR	LR	LR	LR	LR
Harris et al, 2002 [58]	HR	LR	HR	SC	LR	HR
Hintz et al, 2015 [59]	LR	LR	LR	SC	LR	SC
Hoppitt et al, 2014 [60]	LR	LR	LR	LR	LR	LR
Howell et al, 2018 [112]	LR	LR	LR	LR	LR	LR
Kenardy et al, 2003 [61]	LR	LR	LR	SC	LR	SC
King et al, 2015 [62]	LR	LR	LR	SC	LR	SC
Koydemir & Sun-Selisik, 2016 [63]	SC	SC	HR	SC	LR	HR
Kvillemo et al, 2016 [64]	LR	LR	LR	LR	LR	LR
Lee & Jung, 2018 [102]	LR	LR	LR	LR	LR	LR
Levin et al, 2014 [65]	LR	LR	LR	SC	HR	HR
Levin et al, 2016 [66]	LR	LR	LR	LR	LR	LR
Levin et al, 2017 [67]	LR	LR	LR	SC	LR	SC
Lintvedt et al, 2013 [68]	SC	LR	LR	SC	LR	SC
Mailey et al, 2010 [69]	LR	LR	LR	SC	LR	SC
Mak et al, 2015 [70]	LR	LR	LR	SC	LR	SC
Mak et al, 2017 [71]	LR	LR	LR	LR	SC	SC
McCall et al, 2018 [103]	LR	LR	LR	LR	LR	LR
Melnyk et al, 2015 [72]	SC	LR	LR	SC	SC	HR
Mogoaşe, 2013 [104]	LR	LR	LR	LR	LR	LR
Morris et al, 2016 [73]	LR	LR	LR	SC	LR	SC
Mullin et al, 2015 [74]	SC	LR	LR	SC	LR	SC
Musiat et al, 2014 [75]	LR	LR	LR	LR	LR	LR
Nguyen-Feng et al, 2015 [76]	LR	LR	LR	SC	LR	SC
Nguyen-Feng et al, 2016 [77]	LR	LR	LR	LR	SC	SC
Nguyen-Feng et al, 2017 [78]	LR	LR	LR	LR	LR	LR
Nordmo et al, 2015 [79]	SC	LR	LR	LR	LR	SC
Orbach et al, 2007 [80]	LR	LR	SC	LR	SC	SC
Radhu et al, 2012 [81]	LR	LR	LR	SC	LR	SC
Rasanen et al, 2016 [82]	LR	LR	LR	SC	LR	SC
Richards & Timulak, 2013 [83]	LR	SC	SC	LR	HR	HR
Richards et al, 2013 [84]	LR	LR	LR	LR	LR	LR
Richards et al, 2016 [85]	LR	LR	LR	SC	LR	SC
Rose et al, 2013 [105]	SC	LR	LR	LR	LR	SC
Sagon et al, 2018 [106]	SC	LR	LR	LR	LR	SC
Saleh et al, 2018 [107]	SC	LR	LR	LR	LR	SC
Santucci et al, 2014 [86]	LR	LR	LR	LR	LR	LR
Sarniak, 2009 [108]	SC	SC	LR	LR	LR	SC
Seligman et al, 2007 [87]	SC	LR	LR	SC	LR	SC
Stallman et al, 2018 [109]	LR	LR	LR	LR	LR	LR
Taitz, 2011 [110]	SC	SC	SC	LR	LR	HR
Tillfors et al, 2008 [88]	LR	LR	LR	LR	LR	LR
Villani & Riva, 2008 [111]	LR	LR	LR	LR	LR	LR
Yang et al, 2015 [89]	LR	LR	LR	SC	LR	SC

^a^Dmn: domain.

^b^Bias arising from the randomization process.

^c^Bias due to deviations from intended interventions.

^d^Bias due to missing outcome data.

^e^Bias in measurement of the outcome.

^f^Bias in selection of the reported result.

^g^LR: low risk.

^h^SC: some concerns.

^i^HR: high risk.

**Table 2:** Risk of bias for nonrandomized studies.

Authors and year of publication	Dmn^a^ #1^b^	Dmn #2^c^	Dmn #3^d^	Dmn #4^e^	Dmn #5^f^	Dmn #6^g^	Dmn #7^h^	Overall risk
Benton et al, 2016 [113]	MR^i^	LR^j^	SR^k^	LR	SR	SR	LR	SR
Carey et al, 2016 [114]	MR	LR	LR	LR	LR	SR	LR	SR
Finlay-Jones et al, 2016 [115]	MR	LR	LR	LR	MR	SR	LR	SR
Haas et al, 2008 [116]	LR	N/A^l^	N/A	N/A	N/A	N/A	N/A	LR
Horgan et al, 2013 [117]	MR	LR	LR	LR	SR	SR	LR	SR
Kaczmarek et al, 2013 [118]	LR	N/A	N/A	N/A	N/A	N/A	N/A	LR
Kim et al, 2011 [119]	LR	N/A	N/A	N/A	N/A	N/A	N/A	LR
Levin et al, 2015 [120]	MR	LR	LR	LR	MR	SR	LR	SR
Moir et al, 2015 [121]	MR	LR	SR	LR	NI^m^	SR	LR	NI
North et al, 2002 [122]	MR	LR	LR	LR	LR	SR	LR	SR
Palacios et al, 2018 [128]	MR	LR	LR	LR	MR	SR	LR	SR
Sharry et al, 2013 [123]	MR	LR	LR	LR	MR	SR	LR	SR
Spadaro & Hunker, 2016 [124]	MR	LR	LR	LR	LR	SR	LR	SR
Trockel et al, 2011 [125]	CR^n^	LR	LR	LR	SR	SR	LR	SR
Williams et al, 2014 [126]	MR	LR	LR	LR	LR	LR	LR	MR
Wilson et al, 1991 [127]	MR	CR	LR	LR	LR	SR	CR	CR

^a^Dmn: domain.

^b^Bias due to confounding.

^c^Bias in selection of participants into the study.

^d^Bias in classification of interventions.

^e^Bias due to deviations from intended interventions.

^f^Bias due to missing data.

^g^Bias in measurement of outcomes.

^h^Bias in selection of the reported result.

^i^MR: moderate risk.

^j^LR: low risk.

^k^SR: serious risk.

^l^N/A: not applicable. Any study deemed low risk in Domain #1 is considered low risk as a whole; thus, other domains are N/A.

^m^NI: no information.

^n^CR: critical risk.

## Discussion

### Principal Findings

This study aimed to synthesize the literature on the effectiveness, usability, acceptability, uptake, and adoption of digital mental health interventions for (1) treating depression and anxiety and (2) enhancing psychological well-being among college students. In doing so, the types of interventions that have been developed and tested were characterized. The vast majority of included studies reported that the digital mental health interventions of interest were either effective, or partially effective, in producing beneficial changes in the main psychological outcome variables. This is consistent with past meta-analyses on digital mental health programs for college students [17,18] and is consistent with the broader literature on digital mental health interventions [134,135]. Effectiveness did not appear to substantially vary by type of digital mental health intervention, indicating that computer-, Web-, mobile-, and virtual reality–based interventions all hold potential for improving mental health on college campuses.

The majority of programs were studied on university campuses and enrolled broad samples of undergraduate and graduate students. The focus on universities was not surprising, as many studies were conducted at the university with which the researchers were affiliated, likely because of a combination of ease and investment in one’s own community. Fewer studies took place within health professional (eg, medical school and nursing school) programs. It was notable that only 1 study comprised a community college sample, as it is widely recognized that community college students have higher rates of unmet mental health needs compared with students in traditional 4-year colleges and universities [136-138]. Furthermore, community college students are likely to face additional barriers to accessing care as they are more likely to attend school part-time while balancing other responsibilities and commitments, and many community college campuses do not provide mental health services [138]. Thus, this appears to be a priority area for further research and intervention development.

College students are often used as a convenience sample for psychological research [139]. Therefore, to interpret the findings in light of whether the included studies aimed to specifically target students, we examined whether the designs appeared specific to college student mental health. We found that the majority of studies (n=68) were focused explicitly on college student mental health, as opposed to using students for convenience sampling. This majority finding highlights the potential for these programs to be more broadly disseminated and implemented on college campuses.

Similar to what has been observed in digital mental health intervention programs for general adult populations [22,134], there were notable rates of participant attrition and early program discontinuation in many of the studies. An individual may discontinue use of a digital mental health intervention for a variety of factors. These include positive reasons, such as early mood improvements resulting in the individual no longer having a need for the intervention tools. More often though, early discontinuation of such programs appears be the result of an unsatisfying user experience. Although user experience is multifaceted, the core components of user experiences include the program’s usability and acceptability, which were a focus of this study. A recent review of user engagement with mental health apps found that problems emerge because apps (1) are not designed with users in mind, (2) do not address problems users care most about, (3) do not respect user privacy, (4) are not seen as trustworthy, and (5) are unhelpful in emergencies [140]. Although this review focused on mental health apps [140], these themes appear to be translatable to reasons for poor engagement with other types of digital mental health interventions. Indeed, these themes suggest usability problems, which decrease the likelihood of user engagement because of a mismatch of design with user needs [141,142]. The majority of interventions included in this review were unnamed programs developed for research purposes, and although many reported on participant satisfaction with the program, the extent to which the interventions were tested for user experience before these trials remains largely unknown. Utilization of user-centered design and usability testing is a growing practice in digital health interventions for depression [143], smoking cessation [144,145], and diverse patient groups [146]. Indeed, this practice promotes the likelihood that the intervention is appropriately engaging, intuitive to use, and pleasing to the intended user population. Therefore, assessing for usability is a critical component in establishing the feasibility, efficacy, and generalizability of a digital health intervention, particularly for specialty populations [147].

Although user-centered design can produce programs that are more engaging and enjoyable for users, design principles alone are unlikely to produce interventions that are sustainably used on college campuses. The research-to-practice gap for digital mental health interventions is increasingly being recognized, and leaders in the field have proposed strategies to routinely incorporate implementation science methods into the study of digital mental health interventions [148,149]. These models highlight the importance of the systems in which interventions are to be introduced, and although all studies included in this review focused on college students as a population of interest, very few examined college campus systems and tested the implementation of programs onto campuses [86,109,116,119]. Increasingly, calls are being made to collect implementation-relevant data while testing new digital mental health programs or testing existing programs on new populations [150-152]. For digital mental health to fully realize its potential for college students, digital health researchers need to embrace methods and models from implementation science, such as hybrid trial designs [153], and add to the body of knowledge on how to create and support a campus mental health system that actively uses digital mental health programs.

### Strengths and Limitations

This study should be interpreted in light of its strengths and limitations. Consistent with best practices, the articles were reviewed by 2 independent reviewers and risk of bias was assessed. The moderate-to-severe risk of bias found in many of the included randomized and nonrandomized trials indicates that the results reported may be biased in favor of the digital mental health tools and should be evaluated in that context. Bias primarily emerged because the outcomes were self-reported in nature and the participants were aware of the intervention they received—2 issues that are exceedingly common in digital health research. Although the search strategy was developed with an experienced research librarian and an additional handsearch was used, it is possible that some relevant publications were missed in the search. Several reviewed studies used active controls or comparison interventions that produced similar effects to the intervention of interest, so we were unable to evaluate the effectiveness of intervention ingredients to inform what components (eg, features or techniques) are relevant for achieving behavior change. Without the gold standard interventions in digital health for college students that could serve as comparisons with newly developed interventions, several studies that were reviewed used active controls or comparison interventions that produced similar effects to the intervention of interest. In addition, none of the included studies utilized noninferiority analyses. Therefore, the true efficacy of most of the interventions was unclear.

Another strength is that we did not limit this review to RCTs of computer- and Web-based programs. As such, this study expands on past work by offering a much broader look at the types of digital mental health programs that have been available for students and a look at the uptake and adoption of such interventions. Uptake and adoption could not have been meaningfully examined if this review was limited to RCTs. However, the consequence of including multiple trial designs precluded us conducting a meta-analysis because of the heterogeneity of the data included.

### Conclusions

Digital mental health interventions for depression, anxiety, and the enhancement of psychological well-being have the potential to improve the mental health of college students around the world. The majority of interventions have focused on Web-based technologies, and there remains a need for further research on interventions delivered via mobile phones. To date, published studies on digital mental health programs have primarily been focused on establishing efficacy and/or effectiveness rather than on supporting program uptake and adoption across campus communities. For these programs to realize their potential, they need to be successfully and sustainably implemented on college campuses as part of the array of available mental health services. Further research on digital mental health interventions for college students should focus on designing and testing programs that are viewed as usable and acceptable to students and on methods of implementing such programs on college campuses.

## Appendix

Multimedia Appendix 1 Search strategy overview.

Multimedia Appendix 2 Study details.

Multimedia Appendix 3 Study results.

## Abbreviations

PRISMA: Preferred Reporting Items for Systematic Reviews and Meta-Analyses
RCT: randomized controlled trial
