# Supplementary material for: Digital Mental Health Interventions for Depression, Anxiety, and Enhancement of Psychological Well-Being Among College Students: Systematic Review
Source: J Med Internet Res. 2019 Jul 22;21(7):e12869. doi: 10.2196/12869 (PMC6681642; doi:10.2196/12869)
Supplement: Multimedia Appendix 1 [file jmir_v21i7e12869_app1.pdf]

Search Strategy Overview

MEDLINE Ovid search strategy

1. Universities/
2. ((postsecondary or post-secondary or tertiary or undergraduate* or graduate* or colleg* or universit*) adj10 (educat* or school* or student* or person* or people or women or woman or female* or men or man or male* or youth* or classroom* or curricul* or institution* or academ*)).ab,ti.
3. 1 or 2
4. exp Depression/
5. exp Depressive Disorder/
6. exp Anxiety/
7. exp Adjustment Disorders/
8. exp Affective Symptoms/
9. exp Anxiety Disorders/
10. exp Mood Disorders/
11. (depress* or anxiet* or anxious* or dysthymi* or panic* or phobic or phobia* or (mood adj disorder*) or (adjustment adj disorder*) or (affective adj disorder*) or (affective adj symptom*)).ab,ti.
12. 4 or 5 or 6 or 7 or 8 or 9 or 10 or 11
13. exp Telemedicine/
14. exp Computer Systems/
15. Communications Media/
16. exp mass media/
17. exp audiovisual aids/
18. exp telecommunications/
19. (android* or app or apps or audio* or blog* or cellphone* or (cell adj phone*) or computer* or cyber* or DVD* or digital* or ehealth or e-health or electronic* or email* or e-mail* or ePortal* or e-Portal* or eRecovery or eTherap* or e-therap* or gaming or (instant adj message*) or internet or ipad* or i-pad* or iphone* or i-phone* or ipod* or i-pod* or Kindle* or laptop* or mhealth or m-health or mobile or multimedia or multi-media or oCBT or online or on-line or podcast* or remote* or SMS or (smart adj phone*) or (social adj network*) or software or telephone or smartphone* or technolog* or telecomm* or telehealth* or telemed* or telemonitor* or teletherap* or (text adj message*) or texting or video* or virtual* or web or media or wireless or wearable* or Facebook or Instagram or Myspace or Reddit or Skype or Snapchat or Tumblr or Twitter or WhatsApp or YouTube).ab,ti.
20. 13 or 14 or 15 or 16 or 17 or 18 or 19
21. 3 and 12 and 20

Cochrane Library

#1 MeSH descriptor: [Universities] explode all trees
#2 (postsecondary or post-secondary or tertiary or undergraduate* or graduate* or colleg* or universit*) near/10 (educat* or school* or student* or person* or people or women or woman or female* or men or man or male* or youth* or classroom* or curricul* or institution* or academ*):ti,ab,kw (Word variations have been searched)
#3 #1 or #2
#4 MeSH descriptor: [Depression] explode all trees
#5 MeSH descriptor: [Depressive Disorder] explode all trees
#6 MeSH descriptor: [Anxiety] explode all trees
#7 MeSH descriptor: [Adjustment Disorders] explode all trees
#8 MeSH descriptor: [Affective Symptoms] explode all trees
#9 MeSH descriptor: [Anxiety Disorders] explode all trees
#10 MeSH descriptor: [Mood Disorders] explode all trees
#11 depress* or anxiet* or anxious* or dysthymi* or panic* or phobic or phobia* or (mood next disorder*) or (adjustment next disorder*) or (affective next disorder*) or (affective next symptom*):ti,ab,kw (Word variations have been searched)
#12 #4 or #5 or #6 or #7 or #8 or #9 or #10 or #11
#13 MeSH descriptor: [Telemedicine] explode all trees
#14 MeSH descriptor: [Computer Systems] explode all trees
#15 MeSH descriptor: [Communications Media] this term only
#16 MeSH descriptor: [Mass Media] explode all trees
#17 MeSH descriptor: [Audiovisual Aids] explode all trees
#18 MeSH descriptor: [Telecommunications] explode all trees
#19 android* or app or apps or audio* or blog* or cellphone* or (cell next phone*) or computer* or cyber* or DVD* or digital* or ehealth or e-health or electronic* or email* or e-mail* or ePortal* or e-Portal* or eRecovery or eTherap* or e-therap* or gaming or (instant next message*) or internet or ipad* or i-pad* or iphone* or i-phone* or ipod* or i-pod* or Kindle* or laptop* or mhealth or m-health or mobile or multimedia or multi-media or oCBT or online or on-line or podcast* or remote* or SMS or (smart next phone*) or (social next network*) or software or telephone or smartphone* or technolog* or telecomm* or telehealth* or telemed* or telemonitor* or teletherap* or (text next message*) or texting or video* or virtual* or web or media or wireless or wearable* or Facebook or Instagram or Myspace or Reddit or Skype or Snapchat or Tumblr or Twitter or WhatsApp or YouTube:ti,ab,kw (Word variations have been searched)
#20 #13 or #14 or #15 or #16 or #17 or #18 or #19
#21 #3 and #12 and #20

Embase

#20) #19 AND ('article'/it OR 'article in press'/it OR 'review'/it)
#19) #3 AND #11 AND #18
#18) #12 OR #13 OR #14 OR #15 OR #16 OR #17
#17) android*:ab,ti OR app:ab,ti OR apps:ab,ti OR audio*:ab,ti OR blog*:ab,ti OR cellphone*:ab,ti OR 'cell phone':ab,ti OR 'cell phones':ab,ti OR computer*:ab,ti OR cyber*:ab,ti OR dvd*:ab,ti OR digital*:ab,ti OR ehealth:ab,ti OR 'e health':ab,ti OR electronic*:ab,ti OR email*:ab,ti OR 'e mail*':ab,ti OR eportal*:ab,ti OR 'e portal*':ab,ti OR erecovery:ab,ti OR etherap*:ab,ti OR 'e therap*':ab,ti OR gaming:ab,ti OR 'instant message':ab,ti OR 'instant messages':ab,ti OR 'instant messaging':ab,ti OR internet:ab,ti OR ipad*:ab,ti OR 'i pad*':ab,ti OR iphone*:ab,ti OR 'i phone*':ab,ti OR ipod*:ab,ti OR 'i pod*':ab,ti OR kindle*:ab,ti OR laptop*:ab,ti OR mhealth:ab,ti OR 'm health':ab,ti OR mobile:ab,ti OR multimedia:ab,ti OR 'multi media':ab,ti OR ocbt:ab,ti OR online:ab,ti OR 'on line':ab,ti OR podcast*:ab,ti OR remote*:ab,ti OR sms:ab,ti OR 'smart phone':ab,ti OR 'smart phons':ab,ti OR 'social network':ab,ti OR 'social networks':ab,ti OR software:ab,ti OR telephone:ab,ti OR smartphone*:ab,ti OR technolog*:ab,ti OR telecomm*:ab,ti OR telehealth*:ab,ti OR telemed*:ab,ti OR telemonitor*:ab,ti OR teletherap*:ab,ti OR 'text message':ab,ti OR 'text messaging':ab,ti OR texting:ab,ti OR video*:ab,ti OR virtual*:ab,ti OR web:ab,ti OR media:ab,ti OR wireless:ab,ti OR wearable*:ab,ti OR facebook:ab,ti OR instagram:ab,ti OR myspace:ab,ti OR reddit:ab,ti OR skype:ab,ti OR snapchat:ab,ti OR tumblr:ab,ti OR twitter:ab,ti OR whatsapp:ab,ti OR youtube:ab,ti
#16) 'telecommunication'/exp
#15) 'audiovisual aid'/exp
#14) 'mass medium'/exp
#13) 'computer system'/exp
#12) 'telemedicine'/exp
#11) #4 OR #5 OR #6 OR #7 OR #8 OR #9 OR #10
#10) depress*:ab,ti OR anxiet*:ab,ti OR anxious*:ab,ti OR dysthymi*:ab,ti OR panic*:ab,ti OR phobic:ab,ti OR phobia*:ab,ti OR 'mood disorder':ab,ti OR 'mood disorders':ab,ti OR 'adjustment disorder':ab,ti OR 'adjustment disorders':ab,ti OR 'affective disorder':ab,ti OR 'affective disorders':ab,ti OR 'affective symptom':ab,ti OR 'affective symptoms':ab,ti
#9) 'mood disorder'/exp
#8) 'anxiety disorder'/exp
#7) 'emotional disorder'/exp
#6) 'adjustment disorder'/exp
#5) 'anxiety'/exp
#4) 'depression'/exp
#3) #1 OR #2
#2) ((postsecondary NEAR/10
(educat* OR school* OR student* OR person* OR people OR women OR woman OR female* OR men OR manOR male* OR youth* OR classroom* OR curricul* OR institution* OR academ*)):ti,ab) OR (('post secondary' NEAR/10
(educat* OR school* OR student* OR person* OR people OR women OR woman OR female* OR men OR man OR male* OR youth* OR classroom*OR curricul* OR institution* OR academ*)):ti,ab) OR ((tertiary NEAR/10
(educat* OR school* OR student* OR person* OR people OR women OR woman OR female* OR men OR man OR male* OR youth* OR classroom* OR curricul* OR institution* OR academ*)):ti,ab) OR ((undergraduate* NEAR/10
(educat* OR school* OR student* OR person* OR people OR women OR woman OR female* OR men OR man OR male* OR youth* OR classroom* OR curricul* OR institution* OR academ*)):ti,ab) OR ((graduate* NEAR/10
(educat* OR school* OR student* OR person* OR people OR women OR woman OR female* OR men OR

man OR male* OR youth* OR classroom*OR curricul* OR institution* OR academ*)):ti,ab) OR
((colleg* NEAR/10
(educat* OR school* OR student* OR person* OR people OR women OR woman OR female* OR men OR man OR male* OR youth* OR classroom* OR curricul* OR institution* OR academ*)):ti,ab) OR
((universit* NEAR/10
(educat* OR school* OR student* OR person* OR people OR women OR woman OR female* OR men OR manOR male* OR youth* OR classroom* OR curricul* OR institution* OR academ*)):ti,ab)
#1) 'university'/exp

Web of Science

Web of Science

5 #3 AND #2 AND #1
Refined by: DOCUMENT TYPES: (ARTICLE OR BOOK CHAPTER OR CORRECTION OR BOOK OR REVIEW)
Indexes=SCI-EXPANDED, SSCI, A&HCI, CPCI-S, CPCI-SSH, BKCI-S, BKCI-SSH, ESCI, CCR-EXPANDED, IC
Timespan=All years

4 #3 AND #2 AND #1
Indexes=SCI-EXPANDED, SSCI, A&HCI, CPCI-S, CPCI-SSH, BKCI-S, BKCI-SSH, ESCI, CCR-EXPANDED, IC
Timespan=All years

3 **TOPIC:** (android* OR app OR apps OR audio* OR blog* OR cellphone* OR "cell phone" OR "cell phones" OR computer* OR cyber* OR dvd* OR digital* OR ehealth OR e-health OR electronic* OR email* OR e-mail* OR eportal* OR e-portal* OR erecovery OR etherap* OR e-therap* OR gaming OR "instant message" OR "instant messages" OR "instant messaging" OR internet OR ipad* OR i-pad* OR iphone* OR i-phone* OR ipod* OR i-pod* OR kindle* OR laptop* OR mhealth OR m-health OR mobile OR multimedia OR multi-media OR ocbt OR online OR on-line OR podcast* OR remote* OR sms OR “smart phone” OR “smart phons” OR “social network” OR “social networks” OR software OR telephone OR smartphone* OR technolog* OR telecomm* OR telehealth* OR telemed* OR telemonitor* OR teletherap* OR “text message” OR “text messaging” OR texting OR video* OR virtual* OR web OR media OR wireless OR wearable* OR facebook OR instagram OR myspace OR reddit OR skype OR snapchat OR tumblr OR twitter OR whatsapp OR youtube)
Indexes=SCI-EXPANDED, SSCI, A&HCI, CPCI-S, CPCI-SSH, BKCI-S, BKCI-SSH, ESCI, CCR-EXPANDED, IC
Timespan=All years

2 **TOPIC:** (depress* or anxiet* or anxious* or dysthymi* or panic* or phobic or phobia* or (mood NEAR/0 disorder*) or (adjustment NEAR/0 disorder*) or (affective NEAR/0 disorder*) or (affective NEAR/0 symptom*))
Indexes=SCI-EXPANDED, SSCI, A&HCI, CPCI-S, CPCI-SSH, BKCI-S, BKCI-SSH, ESCI, CCR-EXPANDED, IC
Timespan=All years

1 **TOPIC:** ((postsecondary or post-secondary or tertiary or undergraduate* or graduate* or colleg* or universit*) NEAR/10 (educat* or school* or student* or person* or people or women or woman or female* or men or man or male* or youth* or classroom* or curricul* or institution* or academ*))
Indexes=SCI-EXPANDED, SSCI, A&HCI, CPCI-S, CPCI-SSH, BKCI-S, BKCI-SSH, ESCI, CCR-EXPANDED, IC
Timespan=All years

PsycINFO

S11	S3 AND S6 AND S9
S10	S3 AND S6 AND S9
S9	S7 OR S8
S8	TI (android* OR app OR apps OR audio* OR blog* OR cellphone* OR (cell W phone*) OR computer* OR cyber* OR DVD* OR digital* OR ehealth OR e-health OR electronic* OR email* OR e-mail* OR ePortal* OR e-Portal* OR eRecovery OR eTherap* OR e-therap* OR gaming OR (instant W message*) OR internet OR ipad* OR i-pad* OR iphone* OR i-phone* OR ipod* OR i-pod* OR Kindle* OR laptop* OR mhealth OR m-health OR mobile OR multimedia OR multi-media OR oCBT OR online OR on-line OR podcast* OR remote* OR SMS OR (smart W phone*) OR (social W network*) OR software OR telephone OR smartphone* OR technolog* OR telecomm* OR telehealth* OR telemed* OR telemonitor* OR teletherap* OR (text W message*) OR texting OR video* OR virtual* OR web OR media OR wireless OR wearable* OR Facebook OR Instagram OR Myspace OR Reddit OR Skype OR Snapchat OR Tumblr OR Twitter OR WhatsApp OR YouTube) OR AB (android* OR app OR apps OR audio* OR blog* OR cellphone* OR (cell W phone*) OR computer* OR cyber* OR DVD* OR digital* OR ehealth OR e-health OR electronic* OR email* OR e-mail* OR ePortal* OR e-Portal* OR eRecovery OR eTherap* OR e-therap* OR gaming OR (instant W message*) OR internet OR ipad* OR i-pad* OR iphone* OR i-phone* OR ipod* OR i-pod* OR Kindle* OR laptop* OR mhealth OR m-health OR mobile OR multimedia OR multi-media OR oCBT OR online OR on-line OR podcast* OR remote* OR SMS OR (smart W phone*) OR (social W network*) OR software OR telephone OR smartphone* OR technolog* OR telecomm* OR telehealth* OR telemed* OR telemonitor* OR teletherap* OR (text W message*) OR texting OR video* OR virtual* OR web OR media OR wireless OR wearable* OR Facebook OR Instagram OR Myspace OR Reddit OR Skype OR Snapchat OR Tumblr OR Twitter OR WhatsApp OR YouTube)
S7	(DE "Telemedicine" OR DE "Computer Usage" OR DE "Computers" OR DE "Computer Games" OR DE "Digital Computers" OR DE "Microcomputers" OR DE "Computer Games" OR DE "Digital Computers" OR DE "Computer Applications" OR DE "Artificial Intelligence" OR DE "Computer Assisted Diagnosis" OR DE "Computer Assisted Instruction" OR DE "Computer Assisted Testing" OR DE "Computer Assisted Therapy" OR DE "Computer Simulation" OR DE "Electronic Learning" OR DE "Computer Software" OR DE "Mobile Devices" OR DE "Cellular Phones" OR DE "Communications Media" OR DE "Audiovisual Communications Media" OR DE "Mass Media" OR DE "Multimedia" OR DE "Social Media" OR DE "Telecommunications Media" OR DE "Films" OR DE "News Media" OR DE "Radio" OR DE "Television" OR DE "Audiovisual Communications Media" OR DE "Online Social Networks" OR DE "Telecommunications Media" OR DE "Telephone Systems" OR DE "Computer Mediated Communication" OR DE "Blog" OR DE "Electronic Communication" OR DE "Text Messaging" OR DE "Educational Audiovisual Aids" OR DE "Internet" OR DE "Online Therapy" OR DE "Teleconferencing")
S6	S4 OR S5
S5	TI (depress* OR anxiet* OR anxious* OR dysthymi* OR panic* OR phobic OR phobia* OR (mood W disorder*) OR (adjustment W disorder*) OR (affective W disorder*) OR (affective W symptom*)) OR AB (depress* OR anxiet* OR anxious* OR dysthymi* OR

panic* OR phobic OR phobia* OR (mood W disorder*) OR (adjustment W disorder*) OR (affective W disorder*) OR (affective W symptom*))

S4 (((DE "Major Depression" OR DE "Affective Disorders" OR DE "Bipolar Disorder" OR DE "Disruptive Mood Dysregulation Disorder" OR DE "Major Depression" OR DE "Mania" OR DE "Seasonal Affective Disorder" OR DE "Dysthymic Disorder" OR DE "Depression (Emotion)" OR DE "Seasonal Affective Disorder" OR DE "Anxiety" OR DE "Computer Anxiety" OR DE "Mathematics Anxiety" OR DE "Performance Anxiety" OR DE "Social Anxiety" OR DE "Speech Anxiety" OR DE "Test Anxiety" OR DE "Anxiety Disorders" OR DE "Acute Stress Disorder" OR DE "Generalized Anxiety Disorder" OR DE "Obsessive Compulsive Disorder" OR DE "Panic Disorder" OR DE "Phobias" OR DE "Post-Traumatic Stress" OR DE "Posttraumatic Stress Disorder" OR DE "Separation Anxiety Disorder" OR DE "Adjustment Disorders") OR (DE "Affective Disorders" OR DE "Bipolar Disorder" OR DE "Disruptive Mood Dysregulation Disorder" OR DE "Major Depression" OR DE "Mania" OR DE "Seasonal Affective Disorder")

S3 S1 OR S2

S2 TI ((postsecondary or post-secondary or tertiary or undergraduate* or graduate* or colleg* or universit*) N10 (educat* or school* or student* or person* or people or women or woman or female* or men or man or male* or youth* or classroom* or curricul* or institution* or academ*)) OR AB ((postsecondary or post-secondary or tertiary or undergraduate* or graduate* or colleg* or universit*) N10 (educat* or school* or student* or person* or people or women or woman or female* or men or man or male* or youth* or classroom* or curricul* or institution* or academ*))

S1 (DE "Colleges" OR DE "Community Colleges") AND (DE "Higher Education" OR DE "Graduate Education" OR DE "Postgraduate Training" OR DE "Undergraduate Education" OR DE "Graduate Education" OR DE "Dental Education" OR DE "Graduate Psychology Education" OR DE "Medical Education" OR DE "Postgraduate Training" OR DE "Clinical Psychology Graduate Training" OR DE "Medical Residency" OR DE "Graduate Schools" OR DE "Professional Specialization")
